# Efficacy and safety of acupuncture treatment for idiopathic deafness: a retrospective study

**DOI:** 10.3389/fneur.2025.1689472

**Published:** 2025-12-05

**Authors:** Chun-li Wen, Yong-kang Zhang, Min Zhang, Li-juan Song, Ling-qun Zhu, Fu-Peng Zhang

**Affiliations:** 1 Department of Traditional Chinese Medicine Shanxi Provincial People's Hospital, The Fifth Hospital of Shanxi Medical University, Taiyuan, China; 2 Shanxi University of Chinese Medicine, Jinzhong, China; 3 Key Laboratory of Chinese Internal Medicine of Educational Ministry and Beijing, Dongzhimen Hospital, Beijing University of Chinese Medicine, Beijing, China

**Keywords:** acupuncture treatment, idiopathic deafness, efficacy, safety, retrospective study

## Abstract

**Objective:**

The aim of this study was to observe the clinical efficacy and safety of conventional treatment combined with acupuncture in the treatment of idiopathic deafness.

**Methods:**

This retrospective study enrolled 141 patients with idiopathic deafness hospitalized from January 2018 to December 2019. We compared the patients according to previous therapy (conventional treatment and acupuncture treatment), demographics, type of deafness, pure tone hearing threshold score, concomitant symptoms, adverse events and outcomes.

**Results:**

A total of 141 patients were assigned to a control group (n = 67, with simple routine Western medicine treatment) and an observation group (n = 74, with acupuncture treatment on the basis of Western medicine therapy). The total effective rate was 98.65% in the observation group, compared with 74.63% in the control group (*p* < 0.001). The tinnitus and vertigo were both improved in both groups, and the observation group was better than the control group (*p* < 0.05). Adverse reactions occurred in one patient (1.35%) in the observation group and in five patients (7.46%) in the control group (*p* = 0.085).

**Conclusion:**

Among patients with idiopathic deafness who can be treated after the onset of symptoms, the combination of acupuncture and conventional treatment is superior to conventional treatment alone for improving clinical efficacy, reducing concomitant symptoms and not increasing the risk of adverse events.

## 1 Introduction

Idiopathic deafness, also known as sudden sensorineural hearing loss (SSNHL), is characterized by a ≥ 30 dB sensorineural hearing loss across at least three contiguous frequencies within 72 h. its incidence ranges from 5 to 150/100,000 individuals, predominantly affecting those aged 41–55 years (1). Based on the frequency and degree of hearing loss, SSNHL can be classified into high-frequency, low-frequency, flat, and total (including extremely severe) hearing loss types (2). The etiology remains unclear, with potential mechanisms involving viral infection, chronic inflammation, immune dysfunction, and vascular microcirculation impairment (3). However, most cases have no identifiable cause.

Systemic and local corticosteroids remain the most widely accepted therapies, as recommended by Chinese and American clinical guidelines (1, 2). Glucocorticoids improve hearing by suppressing inflammation and immune responses, but their efficacy is limited (total effective rate≈60%) (4, 5). Moreover, outcomes depend on factors such as dosage, treatment duration, and administration route, and therapy may cause systemic or local adverse effects (6–8). The vascular disorder hypothesis, supported by imaging evidence (9, 10), suggests that improving inner ear microcirculation could be beneficial (10), yet excessive vasodilation may reduce inner ear perfusion (11). Hyperbaric oxygen therapy can enhance oxygen delivery and has shown hearing improvement when combined with other treatments (12, 13). Additionally, microcirculatory disturbances and viral infections may lead to neural degeneration, forming the rationale for neurotrophic therapy (14–16). Despite these approaches, current pharmacologic treatments remain suboptimal, and safer, more effective therapies are still needed.

In China, the primary treatment for idiopathic deafness is to improve the inner ear and trophic nerve microcirculation through hormone use, hyperbaric oxygen therapy, and traditional Chinese medicine acupuncture treatment (17, 18). According to clinical reports on the treatment of deafness with traditional Chinese medicine (TCM) in recent years, Chinese medicine, acupuncture and other treatment methods have definite curative effects on the treatment of deafness, and most of the curative effects are better than those of simple Western medicines (19). In particular, acupuncture treatment of deafness has become increasingly widely used every year, and it has achieved satisfactory results. Acupuncture therapy has been accepted by the majority of patients for its advantages of lacking adverse reactions, as well as its simplicity, low cost and accurate curative effect (20). The results of the meta-analysis (21) showed that the acupuncture treatment group (acupuncture combined with Chinese and Western medicine, hyperbaric oxygen or simple acupuncture treatment) was superior to the simple drug group (traditional Chinese medicine or Western medicine or hyperbaric oxygen). Among these, combined acupuncture and Western medicine therapy is most frequently used in clinical practice, and simple acupuncture alone may still outperform Western medicine alone (22).

Although previous studies have shown promising results for acupuncture in treating idiopathic deafness, most focused on hearing recovery, while accompanying symptoms such as tinnitus and vertigo have been largely overlooked. These symptoms substantially affect patients’ quality of life and may persist despite hearing improvement. Therefore, a comprehensive evaluation addressing both auditory recovery and associated symptoms is clinically necessary but insufficiently explored. To address this gap, the present retrospective study evaluated the efficacy and safety of acupuncture combined with Western medicine in patients with idiopathic deafness and its accompanying symptoms. The study analyzed hearing recovery, tinnitus and vertigo grading, treatment responses, pure-tone audiometry results, adverse events, and recurrence outcomes.

## 2 Materials and methods

### 2.1 Subjects

This retrospective study enrolled 141 patients with idiopathic deafness in the Department of Traditional Chinese Medicine (TCM) and Department of Ear Nose Throat (E.N.T.) of Shanxi Provincial People’s Hospital from January 2018 to December 2019. This period was selected because it represents the most recent two-year interval with complete inpatient data and adequate follow-up information available for all included patients. Data integrity and completeness were verified through electronic medical record review and follow-up confirmation to ensure representativeness and reliability.

Patients were assigned to the control or observation group according to whether they received a 14-day acupuncture intervention; therefore, this was a non-randomized controlled retrospective study.

Potential confounding factors were controlled through several measures: (1) strict diagnostic, inclusion, and exclusion criteria were applied; (2) baseline characteristics—including age, sex, disease duration, type of deafness, pre-treatment tinnitus grade, pre-treatment vertigo grade, and baseline pure-tone hearing threshold—did not differ significantly between groups (*p* > 0.05); and (3) multivariate regression analysis further confirmed the consistency of the adjusted results with univariate findings.

Outcome assessment for tinnitus and vertigo was conducted by independent evaluators who were blinded to group allocation, and all evaluators were not members of the study team. Therefore, the potential for performance and detection bias was minimized.

Ethical approval was obtained from the Ethics Committee of Shanxi Provincial People’s Hospital, and written informed consent was obtained from all patients and/or their guardians.

### 2.2 Inclusion and exclusion criteria

#### 2.2.1 Diagnostic criteria

Conformed to the diagnostic criteria in the *Guideline for the diagnosis and treatment of sudden deafness* (2): (1) sudden and nonfluctuating sensorineural hearing loss, which might be light, moderate or severe (mostly moderate or severe); (2) unknown causes; (3) might be accompanied by tinnitus, blockage of the ear, and impaired sensation of the skin behind the ear; (4) might be accompanied by vertigo, nausea and vomiting, but not recurrent attacks; and (5) no other cranial nerve damage symptoms other than to cranial nerve VIII.

#### 2.2.2 Inclusion criteria

Patients who (1) met the above disease diagnostic criteria; (2) were aged 18–80, male or female; (3) had no other treatment after the disease onset or with treatments having been stopped for at least 1 week, or with limited treatment effectiveness; (4) had no other serious diseases; (5) were informed of the study and signed the informed consent. Additionally, (6) patients with basic diseases such as hypertension, diabetes and blood diseases were being actively treated and were not excluded from the group as absolute contraindications.

#### 2.2.3 Exclusion criteria

(1) Hearing loss was non-sensorineural, including conductive deafness or mixed deafness caused by an ear foreign body, inflammation, tumor, deformity, trauma, etc. (2) There were obvious infectious diseases before the occurrence of deafness, such as herpes zoster, epidemic cerebrospinal meningitis, mumps, measles, etc. (3) The hearing loss was diagnosed as central deafness, identified by CT, MRI or other relevant examinations; (4) Patients with diabetic neuropathy and diabetic neuropathy basis; (5) There were other clear causes of deafness, such as drug use, noise damage, radiation damage, etc. (6) Patients with a mental illness; (7) Due to other external reasons or individual tolerance, the patients could not promise to adhere to the treatment or follow-up.

#### 2.2.4 Criteria for shedding, removal and suspension

(1) In the course of treatment, if the patients were found to be included incorrectly because they did not meet the inclusion criteria, met the exclusion criteria, or were not treated according to the intervention treatment of this study, they were excluded. (2) Patients lost in follow-up, with poor dependency, or withdrawal without completion of the treatment course would be deemed as falling off. (3) Patients would not continue to participate in the study if there were serious adverse reactions, other serious diseases or other complicating disease during the study.

### 2.3 Treatment methods

All patients were given routine treatment to improve microcirculation and nutrition of nerves: 0.9% normal saline solution 250 mL + *Ginkgo biloba* leaf extract injection (HC20181022 Taiwan Chi Sheng Pharma & Biotech Co., Ltd.) 70 mg IVGTT daily for 14 days; Alprostadil Injection (National Medicine Permission Number H20103100 Xi’an Libang Pharmaceutical Co., Ltd.) 500 μg with small pot daily for 14 days; vitamin B1 injection (National Medicine Permission Number H12020612, Tianjin Jinyao Pharmaceutical Co., Ltd.) 100 mg IM daily for 14 days; Mecobalamine injection (National Medicine Permission Number H20058993 Nanjing Hailing Pharmaceutical Co. LTD, Yangtze River Pharmaceutical Group) 500 μg IM daily for 14 days; 14 days was one course of treatment.

*Control Group:* Given only routine treatment.

*Observation Group:* On the basis of routine treatment, acupuncture therapy was given.

Acupuncture therapy: (1) Acupoint selection: Main acupoint (on the affected side): ErMen (SJ21), TingGong (SI19), TingHui (GB2), YiFeng (SJ17); Adjunct acupoint (on both sides): HeGu (LI4), TaiChong (GB9), Zhongzhu (SJ3); (2) Operation: After routine disinfection by 75% alcohol cotton ball; the 0.35 mm × 40 mm needle (NO: 20150075 Beijing Hanyi Medical Instrument Co., LTD) was used acupuncture by an acupuncturist (All of the acupuncturist had completed a relevant medical course, graduated in the field of acupuncture and have obtained medical practitioner’s certificate. They had all been working for more than 5 years in department of Traditional Chinese Medicine Shanxi Provincial People’s Hospital). After the arrival of the needling sensation, the twirling manipulation was adopted to rotate the needles in each direction 360° for 1 min to promote the needle sensation to the ear. All acupoints were selected and positioned according to GB/T123462006 (23), and the manipulation techniques referred to the research of Academician Shi Xuemin. (3) Frequency and course of treatment: The treatment was performed once every day for 14 days, and the needle remained inserted for 30 min each time, manipulated by hand once every 15 min. The end of a course is the end of the intervention.

### 2.4 Follow-up

All patients were followed up through telephone or WeChat by the same medical staff after 1 week, 1 month and 3 months post discharge, including whether there was recurrence of deafness, tinnitus, vertigo, blockage of the ear and frequency of seizures.

### 2.5 Outcome measures

The therapeutic efficacy was comprehensively assessed based on hearing improvement, relief of accompanying symptoms (tinnitus and vertigo), safety, and recurrence. All assessments were performed independently conducted by two blinded otolaryngologists.

Hearing efficacy was determined by changes in pure-tone audiometric thresholds measured at baseline and 14 days post-treatment, using standard examinations, including pure-tone audiometry, acoustic immittance, auditory brainstem response (ABR), and electrocochleography (ECochG) as indicated. Outcomes were categorized as follows: cure—restoration of hearing thresholds to normal, the level of the healthy ear, or pre-illness state; significant effect—mean improvement ≥30 dB; effective—improvement between 15–30 dB; and invalid—improvement <15 dB.

Tinnitus efficacy was evaluated based on the patient-reported tinnitus grade, which ranged from Level 0 (no tinnitus) to Level 6 (severe, intolerable tinnitus causing suicidal tendency). Intermediate levels included occasional tinnitus without discomfort (Level 1), continuous tinnitus aggravated in quiet environments (Level 2), tinnitus persisting in noisy environments (Level 3), tinnitus associated with attention deficit or sleep disturbance (Level 4), and severe tinnitus affecting work performance (Level 5). Efficacy was defined as: cure—complete resolution; significant effect—improvement ≥2 grades; effective—improvement of 1 grade; and invalid—no change.

Vertigo efficacy was assessed according to the severity of functional impairment in daily life, with grades ranging from Level 0 (no vertigo or complete remission) to Level 5 (inability to perform daily activities without assistance). Intermediate grades were defined as follows: Level 1 (vertigo without functional limitation), Level 2 (temporary interruption of daily activities during attacks with rapid recovery), Level 3 (partial ability to resume daily activities after attacks), and Level 4 (difficulty managing most daily tasks after attacks). The therapeutic outcome for vertigo was quantified using a symptom improvement index:


$$\text{Efficacy index}:[\begin{array}{l} \left(\text{pretreatment score}-\text{posttreatment score}\right)\\ ÷\text{pretreatment score} \end{array}]\times 100\%$$

Accordingly, outcomes were classified as cure ≥90% improvement; significant effect ≥70 and <90%; effective ≥30 and <70%; or invalid <30%.

Safety was monitored throughout the study by documenting adverse events such as headache, earache, palpitations, syncope, bleeding, hypertension, hyperglycemia, and gastrointestinal reactions. Recurrence of hearing loss or accompanying symptoms was assessed during follow-up (Table 1).

**Table 1:** Summary of outcome measures.

Outcome	Assessment criteria	Grading/ Thresholds	Efficacy classification	Formula / Note
Hearing improvement	Change in pure-tone audiometric thresholds (baseline vs. day 14)		Cure: normal/healthy/pre-disease level Significant: ≥30 dB Effective: 15–30 dB Invalid: <15 dB	Pure-tone audiometry, acoustic immittance, ABR, ECochG
Tinnitus severity	Patient-reported symptom grading	Level 0–6 (no tinnitus→ suicidal tendency)	Cure: disappearance Significant: ≥2 grade improvement Effective: 1 grade improvement Invalid: none	—
Vertigo severity	Impact on daily life	Level 0–5 (no vertigo → full dependence)	Cure: efficacy index ≥90% Significant: 70–89% Effective: 30–69% Invalid: <30%	Efficacy index = (pre–post)/pre × 100%
Safety	Adverse events monitoring			Headache, earache, palpitations, etc.
Recurrence	Follow-up evaluation			Relapse of hearing loss or symptoms

### 2.6 Statistical analysis

All statistical analyses were performed using SPSS 26.0 for Windows. Quantitative data with a normal distribution were expressed as the mean ± standard deviation (x̄ ± s), whereas non-normally distributed data was presented as the median and interquartile range (M [P25, P75]). The Kolmogorov–Smirnov test was applied to assess data normality. Qualitative variables were expressed as frequencies or percentages. For group comparisons, one-way ANOVA was used for normally distributed continuous variables, while the Kruskal–Wallis test was used for non-normally distributed data. Categorical variables were compared using the chi-square test.

To control for potential confounding factors, multivariate regression analysis was additionally performed, adjusting for baseline parameters such as age, sex, disease duration, type of deafness, pre-treatment tinnitus grade, pre-treatment vertigo grade, and baseline pure-tone hearing threshold. Since no significant differences were observed between groups for these baseline characteristics, the multivariate regression results were consistent with those of the chi-square test.

All tests were two-sided, with statistical significance set at *p* < 0.05. No multiple-comparison correction (e.g., Bonferroni or false-discovery-rate adjustment) was applied.

## 3 Results

### 3.1 Baseline characteristics

**Figure 1:**
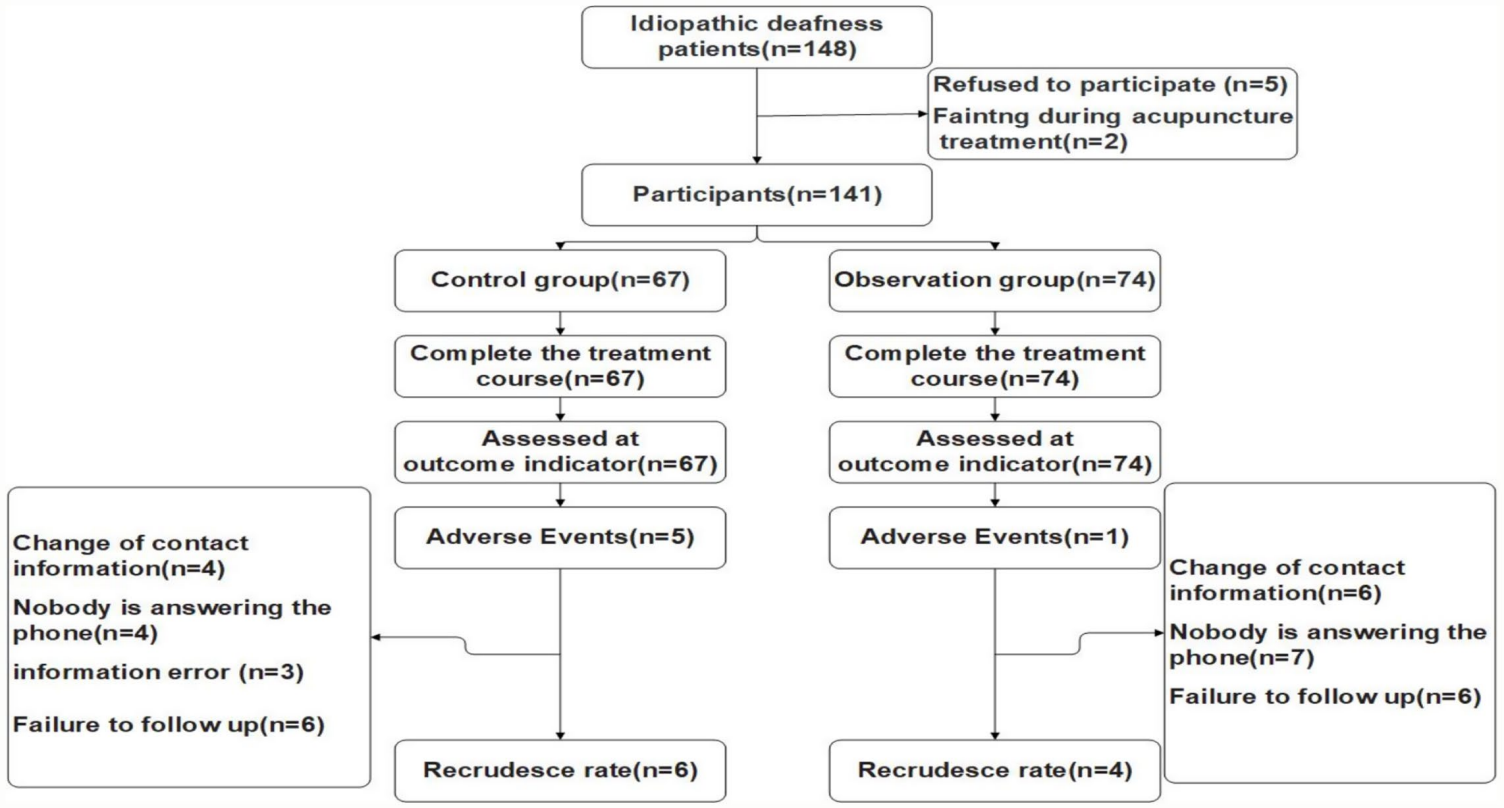
Flow of participants.

A total of 148 patients with idiopathic deafness were admitted to the Department of Traditional Chinese Medicine and E.N.T. department of Shanxi People’s Hospital from January 2018 to December 2019. Finally, 141 cases were included after signing informed consent according to the inclusion and exclusion criteria, treatment course and shedding criteria (Figure 1). The participants were divided into 2 groups according to whether to use acupuncture treatment. The control group included 67 patients: 39 males and 28 females aged 8–75 years, with an average age of 47.7 ± 17.0 years. The course of disease was 1–60 d, with an average of 15.79 ± 16.14 days. In the observation group, there were 74 patients: 43 males and 30 females, aged 19–87 years with an average of 55.6 ± 14.6 years old, and the course of disease ranged from 1 to 60 days with an average of 14.12 ± 15.60 days. There were no statistically significant differences between the two groups in terms of sex, age, course of disease, type of deafness, classification of tinnitus and vertigo or pure tone hearing threshold before treatment (*p* > 0.05, Table 2).

**Table 2:** General characteristics of the participants in the two groups before treatment.

Characteristics	Control group (*n* = 67)	Observation group (*n* = 74)	*p* value
Gender			
Male [cases (%)]	39 (58.21%)	43 (58.11%)	0.990
Female [cases (%)]	28 (41.79%)	31 (41.89%)	
Age (Year, x̄±s)	47.45 ± 17.12	55.61 ± 14.73	0.558
Course of the disease [Day, M(P25, P75)]	10 (4, 21)	7 (3, 20)	0.589
The type of deafness before treatment			
Low frequency group [cases (%)]	6 (8.96%)	13 (17.57%)	0.266
High frequency group [cases (%)]	29 (43.28%)	28 (37.84%)	
Flat group [cases (%)]	21 (31.34%)	22 (29.73%)	
Total deafness group [cases (%)]	11 (16.42%)	11 (14.86%)	
The classification of tinnitus before treatment	Control group (*n* = 61)	Observation group (*n* = 68)	
Level 1 [cases (%)]	4 (6.55%)	8 (11.77%)	0.406
Level 2 [cases (%)]	22 (36.07%)	19 (27.94%)	
Level 3 [cases (%)]	22 (36.07%)	32 (47.06%)	
Level 4 [cases (%)]	13 (21.31%)	9 (13.23%)	
The classification of vertigo before treatment	Control group (*n* = 31)	Observation group (*n* = 48)	
Level 1 [cases (%)]	7 (22.58%)	4 (8.33%)	0.230
Level 2 [cases (%)]	11 (35.48%)	21 (43.75%)	
Level 3 [cases (%)]	8 (25.81%)	12 (25.00%)	
Level 4 [cases (%)]	5 (16.13%)	11 (22.92%)	
The pure tone hearing threshold before treatment			
Left (dB, x̄±s)	67.69 ± 25.17	67.57 ± 25.63	0.987
Right (dB, x̄±s)	63.66 ± 28.60	61.62 ± 29.02	0.676

The type of deafness low-frequency group (below 1,000 Hz frequency hearing loss, at least 250, 500 Hz hearing loss≥20 dBHL), high-frequency group (above 2000 Hz frequency hearing loss, at least 4,000, 8,000 Hz hearing loss ≥20 dBHL), flat group (all frequency hearing decline, an average auditory threshold of 250–8,000 Hz ≤80 dBHL), total deafness group (all frequency hearing decline, an average auditory threshold of 250–8,000 Hz≥81 dBHL).

### 3.2 Comparison of clinical efficacy

#### 3.2.1 Total effective rate of hearing

The total effective rates of clinical hearing in the observation group and the control group were 98.65 and 74.63%, respectively, and there was a significant difference between the two groups (*p* < 0.05, Table 3).

**Table 3:** Comparison of the total effective rate of hearing between groups (*n*,%).

Effects	Control group (*n* = 67)	Observation group (*n* = 74)	*p* value
Cure	**0 (0.00%)**	**15 (20.27%)**	**0.000**
Significant effect	**7 (10.45%)**	**45 (60.81%)**	
Effective	**43 (64.18%)**	**13 (17.57%)**	
Invalid	**17 (25.37%)**	**1 (1.35%)**	
Total Effective Rate (%) (Cure+Significant effect+Effective)/n*100%	**74.63%**	**98.65%**	

Bold values indicate statistically significant differences between the control and observation groups (*p* < 0.05).

#### 3.2.2 Concomitant symptoms

Among the 141 cases, 129 were accompanied by tinnitus, 61 in the control group and 68 in the observation group. The total effective rates of tinnitus were 75.42 and 100%. Among the 141 cases, 79 cases were accompanied by vertigo, 31 in the control group and 48 in the observation group. The total effective rates of vertigo were 96.77 and 100.00%. The treatment effects of tinnitus and vertigo were significantly different between the two groups (*p* < 0.05) as shown in Table 4.

**Table 4:** Comparison of the effective rate of concomitant symptoms between groups (*n*,%).

Effects	Control group	Observation group	*p* value
Tinnitus	**(*n* = 61)**	**(*n* = 68)**	
Cure	2 (3.28%)	22 (32.35%)	0.000
Significant effect	7 (11.48%)	28 (41.18%)	
Effective	37 (60.65)	18 (26.47%)	
Invalid	15 (24.59%)	0 (0.00%)	
Total Effective Rate (%) (Cure+Significant effect+Effective)/n*100%	**75.42%**	**100.00%**	
Vertigo	**(*n* = 31)**	**(*n* = 48)**	
Cure	5 (16.13%)	14 (29.17%)	0.000
Significant effect	5 (16.13%)	21 (43.75%)	
Effective	20 (64.51%)	13 (27.08%)	
Invalid	1 (3.23%)	0 (0.00%)	
Total Effective Rate (%) (Cure+Significant effect+Effective)/n*100%	**96.77%**	**100.00%**	

Bold values indicate statistically significant differences between the control and observation groups (*p* < 0.05).

#### 3.2.3 Pure tone hearing threshold

Before and after treatment, the average pure tone hearing threshold in the control group through self-control was not significantly different (*p* > 0.05). The average pure tone hearing threshold in the observation group through self-control was significantly different (*p* < 0.05). Furthermore, there was a significant difference between the two groups after treatment (*p* < 0.05) (Table 5).

**Table 5:** Comparison of the average pure tone hearing threshold (x^−^ ± S, dB).

Variables	Control group (*n* = 67)	Observation group (*n* = 74)	*p* value
Before treatment (L)	67.69 ± 25.17	67.57 ± 25.63	
After treatment (L)	62.61 ± 24.45	45.41 ± 20.95	0.000
*p* value	**0.239**	**0.676**	
Before treatment (R)	63.66 ± 28.60	61.62 ± 29.02	
After treatment (R)	57.46 ± 24.93	40.81 ± 20.72	0.000
*p* value	**0.184**	**0.000**	

Bold values indicate statistically significant differences between the control and observation groups (*p* < 0.05).

### 3.3 Adverse events

For adverse events, in the control group, 2 participants had elevated blood glucose, and the blood glucose level decreased after hormone withdrawal; 1 patient had increased dizziness, which improved after treatment with minzhanglang; and 2 patients had elevated blood pressure, which dropped to normal after treatment with Nyforda. One participant in the observation group had palpitations after acupuncture, which did not recur after no special treatment. After the treatment, experimental items to estimate safety, including routine blood tests, routine urine tests, Aspartate Aminotransferase (AST), Alanine Aminotransferase (ALT), Blood Urea Nitrogen (BUN), and Creatinine (Cr), were all within the normal reference ranges. There was no significant difference between the control group and the observation group (*p* > 0.05), but the adverse reactions in the observation group were lower than those in the control group, as shown in Table 6.

**Table 6:** Comparison of adverse event rates between groups (*n*,%).

Groups	*n*	In the treatment	Adverse event rate (%)	*p* value
Control group	67	5	7.46%	0.085
Observation group	74	1	1.35%	

### 3.4 Recrudescence

One week after discharge, there was 1 case of recurrent tinnitus in the control group. One month after discharge, there was 1 case of dizziness in the observation group, but there was no tinnitus or hearing loss. After careful inquiry of the medical history, it was considered a new onset of dizziness, and it was recommended to go to the vertigo clinic for treatment. Three months after discharge, there were 4 cases of hearing loss in the control group and 3 cases of aural fullness in the observation group. There was no residual hearing loss or tinnitus. The recurrence rates in the control group and observation group were not significantly different (*p* > 0.05), as shown in Table 7.

**Table 7:** Comparison of recurrence rate between groups (*n*,%).

Groups	*n*	21d	30d	90d	Recurrence rate (%)	*p* value
Control group	50	1	1	4	12.00%	0.721
Observation group	55	0	1	3	5.40%	

## 4 Discussion

The clinical symptoms of idiopathic deafness are sudden hearing loss, usually accompanied by tinnitus, dizziness, nausea, vomiting and other symptoms. Although idiopathic deafness has a certain tendency of self-healing, and only a few patients can gradually recover their hearing by themselves, if there is no timely treatment, most patients still have left hearing damage or the accompanying symptoms persist, which greatly bothers the patients.

As shown in Tables 3, 5, we found that the total effective rate of clinical hearing combined acupuncture treatment was 98.65%, which was significantly higher than that of the nonacupuncture group. The pure tone hearing threshold after acupuncture treatment was significantly higher than that before treatment. At present, both the 2015 edition of the Chinese guidelines and the 2019 edition of the American guidelines recommend that hormone systemic treatment of idiopathic deafness is preferred, and intracameral injection of hormone therapy in the tympanic cavity is used as remedial treatment (1, 2), but the total effective rate of hormone therapy for deafness was found to be approximately 70% in the current clinical studies (24). Therefore, systemic hormone therapy for deafness still needs to be supported by a large number of clinical studies and evidence-based medical evidence. Intratympanic or postauricular subperiosteal glucocorticoid injection glucocorticoids in the treatment of SSNHL do not increase the prognosis of deafness (8). The 2015 edition of the Chinese guidelines considers that hormones combined with vasoactive drugs and drugs to improve blood rheology in the acute phase of sudden deafness are effective for all types of deafness, which is better than a single drug (2), and the efficacy can reach more than 70% (25). In this study, after the treatment of idiopathic deafness with the combination of acupuncture treatment, the total effective rate of hearing reached 98.65%. The research of Mo Wenquan et al. (26) showed that comprehensive treatment of senile deafness is better than pure acupuncture and pure Western medicine treatment, and the clinical total effective rate reached 82.5%. A number of studies have shown that Western medicine, Chinese medicine, acupuncture, moxibustion, hyperbaric oxygen and other means of comprehensive treatment of deafness are better than single Western medicine treatment. This is consistent with the results of this study (27–30).

As shown in Table 3, the study showed that regardless of the accompanying tinnitus or vertigo symptoms, the total effective rate of clinical hearing was 100% significantly higher than that of the nonacupuncture group after combined acupuncture treatment. The vast majority of current studies have focused on improving hearing. However, for accompanying symptoms, there is no specific treatment in most cases. Studies have shown that acupuncture therapy has a good effect for accompanying symptoms of idiopathic deafness, and it also has some advantages such as simple operation, fewer side effects and quick effect, and the efficacy of acupuncture treatment of accompanying symptoms is better than western medicine treatment alone (31). Acupuncture promotes blood circulation in the ear (32) and reduces inflammatory reactions by enhancing the amount of local stimulus (33), which is advantageous for cochlear neuron function restoration. It can not only improve hearing function but also relieve accompanying symptoms such as tinnitus and so on. The curative effect is more prominent.

In this study, there were 5 cases of adverse reactions in the control group, and 4 cases had elevated blood pressure and blood sugar. The analysis showed that it was related to the use of hormones, and when hormone use was stopped, blood pressure and blood sugar decreased. While the observation group did not use hormone therapy, the occurrence of adverse reactions was lower. Clinically, the treatment of idiopathic deafness with traditional Chinese medicine can reduce the side effects of Western medicine, improve symptoms such as tinnitus and vertigo, and improve the total effective rate. Traditional Chinese medicine treatment can have a long-term effect through the internal overall regulatory mechanism and reduce the recurrence rate to compensate for the lack of Western medicine. Studies have shown that acupuncture has a significant effect on the treatment of idiopathic deafness with a low recurrence rate (34), and the observation group had no recurrence in this study. This reflects the advantages of integrated traditional Chinese and Western medicine in the treatment of idiopathic deafness.

The deficiency of this study lies in the small number of study cases. The correlation between the course of disease and prognosis and the correlation between deafness classification and prognosis have not been studied in depth. For the relevance of the disease course and prognosis and the deafness classification and prognostic relevance, this study did not study in depth. In addition, because the follow-up time was short, the long-term treatment effect, adverse reactions and recurrence rate were not reflected. Due to the small sample size, there is a statistically significant deviation in adverse reactions and recurrence rates. Further exploration is needed after expanding the sample size.

This study has several limitations inherent to its retrospective and non-randomized design. Although baseline characteristics were balanced, the absence of multivariable adjustment for potential confounders—such as specific comorbidities and prior treatments—leaves the possibility of residual confounding. Furthermore, no statistical correction for multiple comparisons was applied, which may increase the risk of Type I error. The generalizability of the findings is constrained by the relatively small sample size and the lack of a prospective power calculation, which also limits the interpretability of subgroup analyses and safety trends. Additionally, the reliance on subjective patient-reported scales for tinnitus and vertigo outcomes, coupled with a limited follow-up period, restricts the objective assessment of symptom evolution and long-term efficacy. Future prospective studies featuring larger cohorts, longer follow-up, objective biomarkers, and pre-specified statistical adjustments are needed to validate these preliminary findings.

## 5 Conclusion

Acupuncture has a definite effect on idiopathic deafness, and the effect is better than that of drug therapy alone. It can not only improve the total hearing efficiency but also greatly reduce the duration of accompanying symptoms and improve the clinical symptoms of patients. At the same time, the incidence of adverse reactions is low, and the risk of recurrence is low, which is worthy of extensive clinical application in the future.

## Data Availability

The original contributions presented in the study are included in the article/supplementary material, further inquiries can be directed to the corresponding authors.
